# Effect of single caffeine intake on neuropsychological functions in healthy volunteers: A double-blind placebo-controlled study

**DOI:** 10.1371/journal.pone.0202247

**Published:** 2018-10-31

**Authors:** Yuki Konishi, Hikaru Hori, Kenta Ide, Asuka Katsuki, Kiyokazu Atake, Ryohei Igata, Takamitsu Kubo, Hirotaka Tominaga, Hiroki Beppu, Toshio Asahara, Reiji Yoshimura

**Affiliations:** 1 Department of Psychiatry, University of Occupational and Environmental Health, Kitakyushu, Japan; 2 Department of Pharmacy, Hospital of the University of Occupational and Environmental Health, Kitakyushu, Japan; University of Bari, ITALY

## Abstract

**Objective:**

We investigated the effects of a single instance of caffeine intake on neurocognitive functions and driving performance in healthy subjects using an established cognitive battery and a driving simulator system.

**Methods:**

This study was conducted in a double-blind, randomized, placebo-controlled manner from February 19, 2016 to August 6, 2016. Caffeine intake was discontinued 3 days prior to the study. Participants were randomly assigned to receive 200-mg doses of caffeine or a placebo. Thirty minutes after administration, cognitive functions were evaluated via the Symbol Digit Coding Test (SDC), the Stroop Test (ST), the Shifting Attention Test (SAT) and the Four Part Continuous Performance Test (FPCPT). After the cognitive function tests were conducted, driving performance was evaluated using a driving simulator. We measured the brake reaction time (BRT) in the Harsh-braking test and the standard deviation of the lateral position (SDLP) in the Road-tracking test.

**Results:**

Of 100 randomized subjects, 50 (50%) of 100 in the caffeine group and 50 (50%) of 100 in the placebo group completed the study. Participants in the caffeine group had more correct responses than participants in the placebo group on the SAT (P = 0.03) and made fewer errors (P = 0.02). Participants in the caffeine group exhibited shorter times in the Harsh-braking test than participants in the placebo group (P = 0.048).

**Conclusions:**

A single instance of caffeine intake changed some neurocognitive functions and driving performance in healthy volunteers.

**Trial registration:**

UMIN000023576.

## Introduction

Caffeine is a widely consumed stimulant that is contained in a variety of foods and beverages, such as coffee, tea, soft drinks, and chocolate, and is typically used as an energy source. An estimated 80% of people in the world consume caffeine-containing beverages each day[1]. Caffeine is known psychoactive stimulant resulting in heightened alertness and arousal and improvement of cognitive performance in short-term effect and decrease the risk of cognitive impairment/decline and dementia/AD later in life[2–7]. Beyond that caffeine has beneficial effects against a few acute and chronic neurological disorders including stroke, Parkinson’s disease, amyotrophic lateral sclerosis, dementia, and Alzheimer’s disease. Caffeine is generally considered a functional or beneficial drug because it can improve mood and alertness at low doses. At high doses, caffeine produces adverse intoxicating effects. Therefore, caffeine consumption is typically self-limiting and compatible with a social and productive life. Caffeine is considered to have a low abuse potential, but perhaps its modest reinforcing effects promote the desirability of food and beverages that already have pleasant flavors and aromas. These caffeinated beverages and foods help us focus our attention and provide energy to continue working. Although the question of whether populations are collectively dependent on caffeine has been raised, coffee drinking is thought to be “more a dedicated habit than a compulsive addiction”[8]. Caffeine has also been shown to be effective in real-life driving studies[9]. Drivers often consume caffeinated beverages and food to overcome sleepiness and/or fatigue[10, 11]. The only molecular targets for caffeine at nontoxic doses are the adenosine receptors in the brain, especially the inhibitory A1 receptors and the facilitory A2A receptors (A2AR)[12]. Caffeine is rapidly absorbed into the body within 45 min[13]. It achieves a peak plasma concentration within 15 to 120 min after intake[14], and its average peak value occurs at 30 min[13]. Its metabolic half-life is 3 to 5 hr[15]. The pharmacological (pharmacodynamic and pharmacokinetic) actions of caffeine might be associated with neurocognitive functions, including driving performance.

To date, few studies have examined the effect of a single instance of caffeine intake on neurocognitive performance. Thus, we investigated this effect, including the effect on driving performance, using an established cognitive battery and a driving simulator system, in healthy Japanese subjects.

## Materials and methods

### Participants

One hundred one healthy Japanese volunteers were enrolled in the present study from February 19, 2016 to August 6, 2016. One participant withdraw consent to the study after enrolled, so finally one hundred participants were analyzed (males/females, 50/50; range 22–59 years). All participants had a valid driver’s license. We confirmed that the participants had not been diagnosed with any psychiatric diseases via the Structured Clinical Interview for DSM-Ⅳ(SCID) at the time of the study. Exclusion criteria consisted of participants with a gastric ulcer, cardiac disorder or glaucoma. Female participants could not be pregnant, nursing or have a possibility of being pregnant. Caffeine intake was stopped for at least 3 days before the test day. This study was approved by the ethics committee at the University of Occupational and Environmental Health(H27-184) on December 9, 2015 and written informed consent was obtained from each subject prior to participation. No changes were made to the study protocol after study commencement. The authors confirm that all ongoing and related trials for this drug/intervention are registered. The reason for the delay in register this study was we had only forgotten about the process. After we noticed that, we had rapidly registered.

### Study design

**Fig 1:**
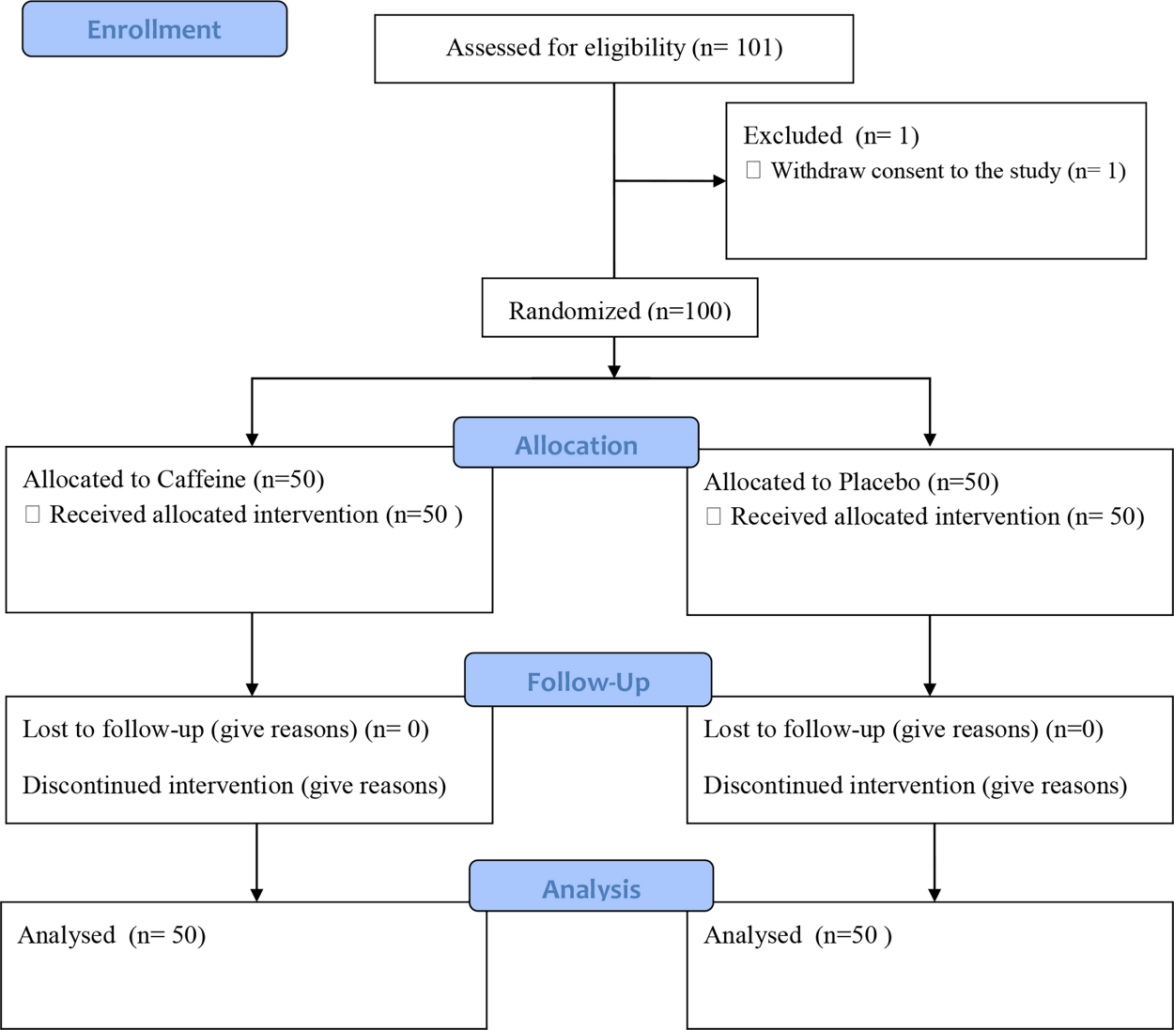
Participants recruitment flowchart.

This study was conducted in a double-blind, randomized, placebo-controlled manner from January 2016 to December 2016 (S1 File). The participants were randomly assigned to either the caffeine group (N = 50) or the placebo group (N = 50) via the envelope method (Fig 1). The protocol (S2 File) was conducted as follows. First, individuals did not consume caffeine for 3 days prior to the study. Participants were randomly assigned to receive 200-mg doses of caffeine or a placebo. Blood samples were obtained before and 120 minutes after the administration of caffeine or a placebo. The plasma caffeine concentration was measured via high-performance liquid chromatography (HPLC). Thirty minutes after administration, the cognitive functions of participants were evaluated with the Symbol Digit Coding Test (SDC), the Stroop Test (ST), the Shifting Attention Test (SAT) and the Four Part Continuous Performance Test (FPCPT) using Cognitrax®, a software program used to test neurocognitive functions. After the cognitive function tests were conducted, the driving performance of participants was evaluated using a driving simulator. We took about 30 minutes to evaluate the cognitive function tests and 50 minutes to evaluate the driving performance. So, Time to check everything was about 2 hours. It was checked at time whenever objects choose, and it wasn't unified. Main outcome measure was driving performance before and after the administration of caffeine or a placebo. Secondary outcome measures were caffeine concentrations and cognitive functions.

### Caffeine and placebo administration

This study was an investigator-initiated, single-center, randomized, double-blind trial of caffeine versus placebo in healthy control at the University of Occupational and Environmental Health. The placebo capsules set in 200mg sugar powder, and the caffeine capsules set in 200mg pure caffeine powder. The number of capsules was kept identical in the placebo trial.

After consent was obtained, the participants were randomized by the specific pharmacist to either caffeine capsules or an identical-looking placebo using envelope method. Double-blinded trial were allocated using sequentially-numbered drug containers. Only the pharmacist was managing it. Therefore, Participants and evaluators were blinded to the identity of the study caffeine and placebo.

### Sample preparation

Blood samples were collected in heparinized tubes and centrifuged for 10 min at 3500 rpm at a controlled temperature of 4°C. The plasma supernatant solution was collected in polypropylene tubes and frozen at -20°C until analysis.

We used 10 μg/mL of 7-β-Hydroxyethyl theophylline as an internal standard (IS). A total of 10 μL of IS working solution and 500 μL of 0.5 M sodium hydroxide were add to 500 μL of the plasma sample in a 1.5 mL microcentrifuge tube. The tube was vortexed for 20 s at 2500 rpm. The liquid was run on a column (Extrelut® NT1 column, Merck Millipore Corporation, Germany) and allowed to stand for 10 min. After standing, 5 mL of dichloromethane was passed through the column, and the extraction liquid was collected in a glass tube. It was evaporated to dryness for approximately 45 min. We added 400 mL of the mobile phase, and the glass tube was vortexed for 20 s at 10,000 rpm.

### Analysis of plasma caffeine levels

**Fig 2:**
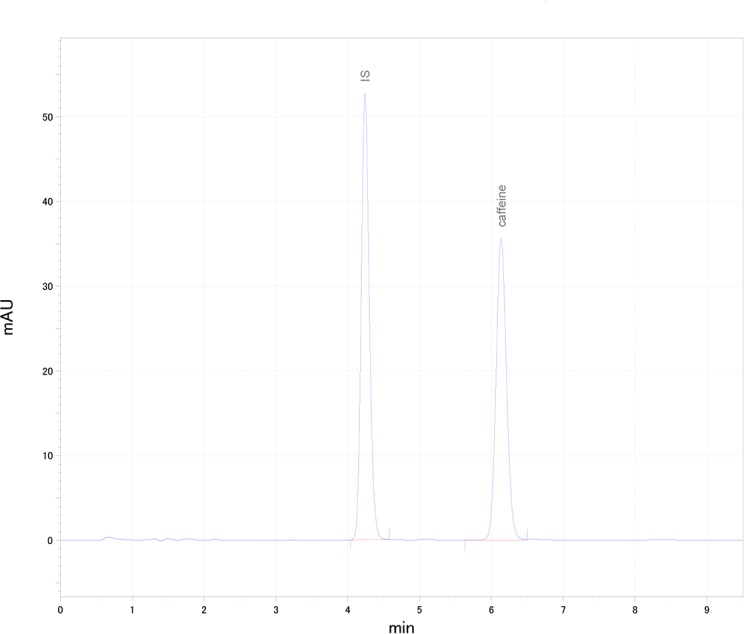
A typical chromatochart of measuring plasma caffeine levels.

Plasma caffeine levels were analyzed using HPLC (Agilent 1220 Infinity II LC, Agilent Technologies, Inc. United States). The HPLC system consisted of a gradient pump, an auto sampler, a column thermostat and a detector sensor with variable wavelengths. A TSK gel ODS-100V (4.6 × 750 mm, 3-μm) column was used. Separation was performed at 40 temperatures. A mobile phase consisting of 0.1% acetic acid, 1 M KH2PO4, 1 mM 1-heptanesulfpnic acid sodium salt and acetonitrile was used at a flow rate of 1.0 mL/min. The injection volume was 80 μL, and UV detection was performed at 260 nm. 7-β-Hydroxyethyl theophylline was used as an IS. It was shows a typical chromatochart of caffeine (Fig 2). The retention time of the IS was approximately 5 min, and that of caffeine was approximately 7 min. The blood level of caffeine was calculated. The determination coefficient of the standard curve was 1.00.

### Driving simulator

The ability to drive was assessed via two driving tasks. A driving simulator (UC-Win/Road Driving Simulator, FORUM8 Co., Ltd. Japan) was used to evaluate driving performance.

**Fig 3:**
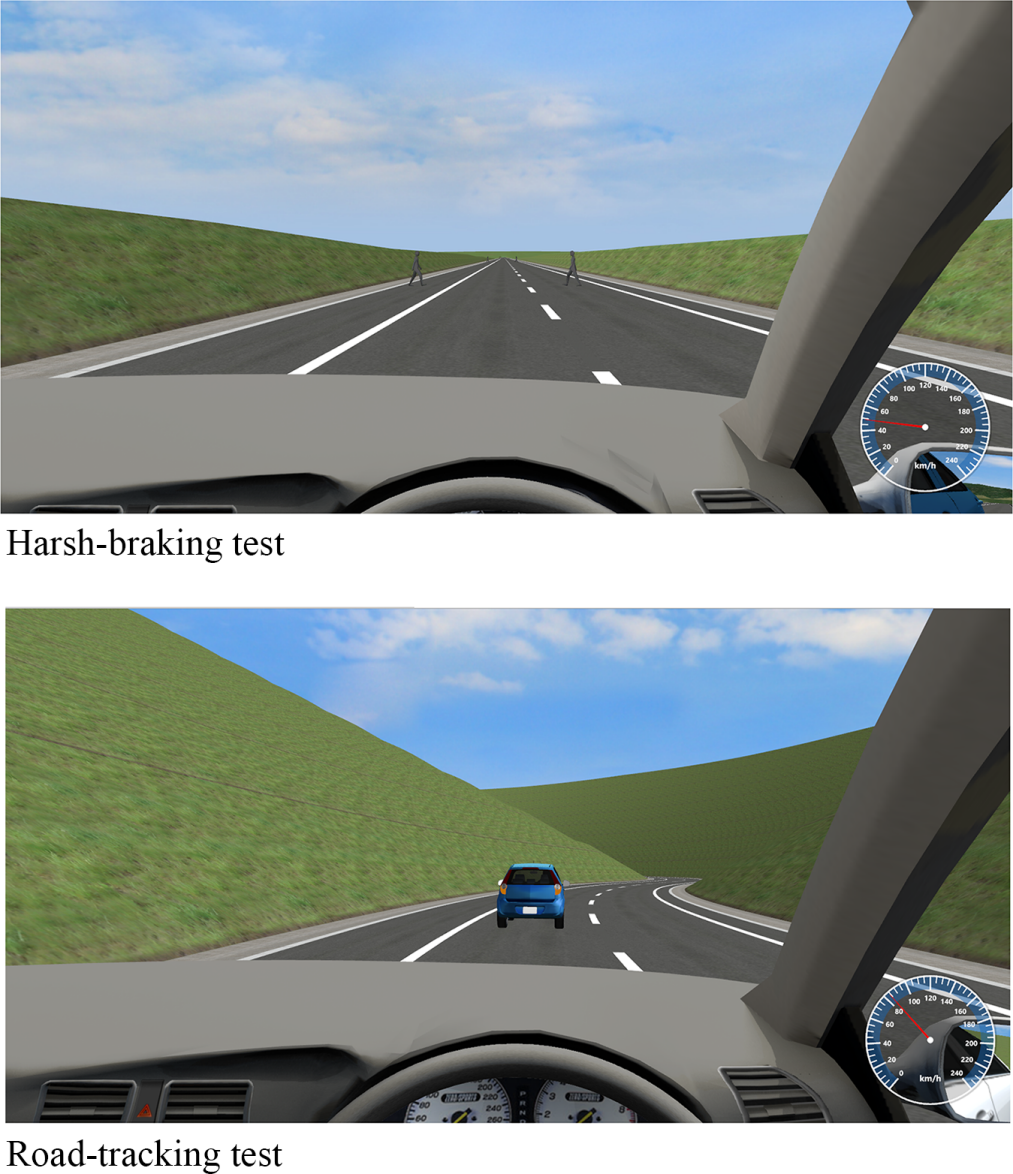
An example scenery of UC-Win/Road Driving Simulator (FORUM8 Co., Ltd. Japan).

The driving simulator was loaded on a personal computer (PC; Windows 7). It consisted of a steering wheel (SENSO-Wheel, SENSODRIVE GmbH. Germany), accelerator and brake system. Images were displayed on 3 ch 32-inch LCD monitors. The driving tasks were performed in a quiet room. It shows two driving tasks (Fig 3).

#### Harsh-braking test

The test course consisted of a straight 2-lane road with no traffic. A total of 14 paired human models appeared in both sides of the left lane every 125 m. The participant maintained a speed of 50 km/h in the left lane. The human models randomly ran onto the road as the participant’s car approached. After the participants realized that the human models had run onto the road, they stopped quickly as possible to avoid hitting the human models. We measured the brake reaction time (BRT; in milliseconds), which was used as a measure of attention efficiency. Each test consisted of 7 BRT trials.

#### Road-tracking test

The test course consisted of a winding 2-lane road with no traffic. The curvature radius of the 200-m course was constant. The participants were directed to maintain a speed of 80 km/h and stabilize the car in the center of the left lane for 5 min. The coordinate data of the car were recorded at least every 10 milliseconds. The lateral position of the car relative to the right and left lanes was derived from the coordinate data. The standard deviation of the lateral position (SDLP; in m) was used to indicate weaving. This test is based on a road-tracking test developed previously[16, 17].

### Cognitive function

Cognitive function was assessed with the CNS Vital Signs (CNSVS) (CNS Vital Signs LLC, Morrisville, USA) computerized battery of tests. The reliability and validity of the CNSVS has been verified, and it is designed to measure cognitive function[18]. The subjects completed four tests in this battery: the SDC, the ST, the SAT and the FPCPT. These tests were chosen because they enable the measurement of performance in different cognitive domains, including reaction time, cognitive flexibility, processing speed, executive function, working memory, and sustained attention.

### Statistical analysis

Statistical analyses were performed using STATA 14 software. Power analysis, with β  =  0.20 and two-tailed α  =  0.05, was based on the pilot studies conducted for the healthy subjects and assessed SDLP, with about three points improve with caffeine. Based on the available pilot data, a projected standard error of the mean difference was assumed to range from equivalent to the mean difference to twice as much as the mean difference, producing a projected sample size of about 100 patients. The distribution of the all data was checked for normality using Kolmogorov-Smirnov test. When the data was regularly distributed, we used t-test, when was not, we used Mann-Whitney U test. All statistical tests were two-tailed, and a P-value below 0.05 was considered significant.

## Results

### Demographic data

No intergroup differences were found for height, body weight, years of education, gender, cigarette smoking, the age at which a driver’s license was obtained, the initiation of driving, the frequency of driving per week, driving time per day, annual mileage, the number of car accidents, the number of traffic offenses and amount of daily caffeine intake (Table 1).

**Table 1:** Demographic data.

Diagnosis of participants	placebo group(N = 50)	caffeine group(N = 50)	p-value
height (cm) ^a^	164.6 ± 9.2	164.9 ± 8.7	0.88
body weight (kg) ^b^	60.1 ± 12.5	59.4 ± 10.8	0.91
year of education (years) ^b^	16.3 ± 2.1	17.3 ± 2.4	0.07
male/female ^c^	24/26	26/24	0.84
smoking/non-smoking ^c^	8/42	7/43	1.00
age of getting a driver license (years) ^b^	20.0 ± 2.4	19.6 ± 1.6	0.65
age of initiation into drive (years) ^b^	21.6 ± 4.4	20.0 ± 3.7	0.33
frequency of driving per week (times) ^b^	5.0 ± 2.4	5.7 ± 2.2	0.11
driving time per day (min)	60.1 ± 37.7	61.0 ± 43.4	0.84
annual mileage (km) ^b^	9105.2 ± 5735.1	8980.2 ± 6626.9	0.70
number of car accident	0.7 ± 0.9	0.9 ± 1.1	0.52
number of car accident of the year	0.1 ± 0.2	0.0 ± 0.1	0.31
number of traffic offence	1.7 ± 1.7	1.5 ± 1.9	0.36
Amount of daily caffeine intake (mg)	198.4 ± 131.0	225.6 ± 149.7	0.45

The data are the mean ± standard deviation.

a: via non-paired t-tests.

b: Mann-Whitney U test.

c: chi-square tests.

### Plasma caffeine concentration

Two-way analysis of variance showed no differences in the mean plasma caffeine concentration values at baseline between the caffeine group and the placebo group (p = 0.67). A significant difference was observed in plasma caffeine concentration 2 hours after caffeine/placebo administration in the two groups (p<0.001) (Table 2).

**Table 2:** Plasma caffeine concentration.

Diagnosis of participants	placebo group(N = 50)	caffeine group(N = 50)	p-value
Pre (μg/ml)	0.20 ± 0.60	0.23 ± 0.80	0.67
After (μg/ml)	0.16 ± 0.46	10.10 ± 3.29	< 0.001

The data are the mean ± standard deviation for skewed variables using Mann-Whitney U test.

### Cognitive function

The results of the SDC, the ST, the SAT and the FPCPT are shown in Table 3. There were no intergroup differences among the SDC, the ST and the FPCPT.

**Table 3:** Result of neurocognitive tests and driving tests.

Neurocognitive tests		placebo group(N = 50)	caffeine group(N = 50)	p-value
SDC	Correct Responses ^b^	66.1 ± 9.7	67.2 ± 11.2	0.74
	Errors ^b^	1.3 ± 1.4	1.5 ± 1.8	0.58
ST	Simple Reaction Time ^b^	320.3 ± 45.3	308.0 ± 40.2	0.09
	Complex Reaction Time Correct ^b^	620.1 ± 68.3	614.1 ± 70.9	0.65
	Stroop Reaction Time Correct ^a^	704.7 ± 81.0	694.8 ± 89.3	0.56
	Stroop commission Errors ^b^	1.0 ± 1.2	0.9 ± 0.8	0.73
SAT	Correct Responses ^b^	52.2 ± 6.4	55.0 ± 5.7	0.03
	Errors ^b^	3.7 ± 2.6	2.5 ± 2.0	0.02
	Correct Reaction Time ^a^	1000.7 ± 127.6	959.5 ± 133.2	0.12
Four Part CPT	Part 1			
	Average Correct Response Time ^b^	334.2 ± 40.0	324.4 ± 46.2	0.09
	Part 2			
	Correct Responses ^b^	6.0 ± 0.1	6.0 ± 0.0	0.32
	Average Correct Response Time ^a^	412.1 ± 50.3	409.2 ± 46.8	0.77
	Incorrect Responses ^b^	0.0 ± 0.2	0.1 ± 0.4	0.39
	Average Incorrect Response Time ^b^	18.3 ± 91.7	32.8 ± 113.7	0.41
	Omission Errors ^b^	0.0 ± 0.1	0.0 ± 0	0.32
	Part 3			
	Correct Responses ^b^	15.8 ± 0.5	15.8 ± 0.5	1.00
	Average Correct Response Time ^b^	473.1 ± 64.9	454.7 ± 63.6	0.15
	Incorrect Responses ^b^	0.1 ± 0.2	0.0 ± 0.1	0.31
	Average Incorrect Response Time ^b^	28.3 ± 121.6	11.0 ± 77.6	0.31
	Omission Errors ^b^	0.2 ± 0.5	0.2 ± 0.5	1.00
	Part 4			
	Correct Responses ^b^	13.1 ± 2.2	13.4 ± 2.3	0.36
	Average Correct Response Time ^a^	640.5 ± 134.6	619.2 ± 135.3	0.43
	Incorrect Responses ^b^	1.0 ± 1.2	1.1 ± 1.1	0.35
	Average Incorrect Response Time ^b^	455.8 ± 444.8	560.4 ± 453.7	0.39
	Omission Errors ^b^	2.9 ± 2.2	2.6 ± 2.3	0.36
Driving tests		placebo group(N = 50)	caffeine group(N = 50)	p-value
SDLP (m) ^a^		0.87 ± 0.27	0.89 ± 0.25	0.75
BRT (sec) ^a^		0.89 ± 0.12	0.84 ± 0.08	0.048

Abbreviations: SDC = Symbol Digit Coding Test, ST = Stroop Test, SAT = Shifting Attention Test, FPCPT = Four Part Continuous Performance Test, BRT = the brake reaction time, SDLP = the standard deviation of the lateral position.

The data are the mean ± standard deviation for skewed variables.

a: via non-paired t-tests.

b: Mann-Whitney U test.

The caffeine group had more correct responses than the placebo group on the SAT (p = 0.03) and made fewer errors (p = 0.02).

### Driving tests

The effect of caffeine consumption on driving performance is shown in Table 3. On the Road-tracking test, no intergroup differences were observed in the SDLP. The caffeine group exhibited a shorter time than the placebo group in the Harsh-braking test (p = 0.048).

## Discussion

The major findings of the present study included the results of the SAT and Harsh-braking tests. The SAT evaluates the precision and time when subjects notice a change in regularity and is considered to reflect executive functions.

Using the Cognitrax system, executive function was derived from the SAT task by subtracting the number of errors from the correct responses. Cognitive flexibility was derived from the SAT task and the Stroop task by subtracting the number of errors on the SAT task and the Stroop task from the correct responses on the SAT task.

The Harsh-braking test measured braking reaction time. Brake reaction speed was evaluated with a computer-driven device that separately measured the initial reaction speed (IRS) (from the time the light turned red until the foot was removed foot from the accelerator) and the physical response speed (PRS) (removing the foot from the accelerator to full brake depression). A reduced IRS was related to low scores on cognitive factors and missing points in the visual field. A decreased PRS was associated with having three or more physical complaints associated with the legs and feet and having poor vision, which was not related to the PRS; only the IRS depends on vision[19].

The Harsh-braking test evaluates brake reaction time when a pedestrian emerges, which reflects cognitive psychomotor performance, including attention. The Harsh-braking test is considered to reflect visual attention. Few studies have investigated the effects of a single instance of caffeine intake on executive functions[20]. Little definitive information is available regarding the effect of caffeine on the ability to resolve cognitive conflicts[21], inhibit automatic or impulsive responses[22], plan strategic actions, and react to changing circumstances with flexibility. Few studies found that a single instance of caffeine intake did not improve executive functions[23–25]. However, a low dose of caffeine enhanced executive functions on the Jansari Assessment of Executive Functions (JEF) task[26].

The driving test results of the present study suggest that a single instance of caffeine intake improves executive functions but not visual attention. Therefore, whether a single instance of caffeine intake influences executive functions remains controversial. Evaluation of the effects of caffeine on executive functions was difficult because of methodological problems. The habitual use of caffeine might influence the results. We could not definitely conclude that a single instance of caffeine intake directly enhances executive functions, because of not evaluating this point in the present study. Caffeine generally improves vigilance and attention in moderate doses (100–300 mg/day)[8]. The SAT can also evaluate vigilance and attention as well as executive functions. Therefore, it is reasonable to consider that a single instance of caffeine intake enhances executive functions by raising alertness via influencing vigilance and attention. In summary, caffeine intake influenced cognitive functions, including vigilance, attention, and executive functions.

Another finding of the present study was that a single instance of caffeine intake significantly shortened the response time in the Harsh-braking test, which is consistent with the finding that caffeine increases mental arousal[27]. A driver’s recognition and error in judgment are factors associated with a traffic accident. Each recognition range, such as intelligence, attention, concentration, visual-spatial ability and execution function, relates to driving performance when driving on the road and when using a driving simulator[28]. Drivers who consumed caffeinated substances for this purpose had a 63% lower likelihood of an accident than drivers who did not consume caffeinated substances[29]. Caffeine has been shown to improve performance and decrease participant sleepiness during driving and in a driving simulator in other studies[30–32]. The outcome measure of lane maintenance, SDLP, which has been used in many preceding studies, was used to measure driving performance. Mets et al.[33, 34] used SDLP. and Adi et al.[35] used the root mean square (RMS) of the lane position, which is an equivalent method to the SDLP, to measure driving performance. Pierre et al.[10] measured the number of lane crossings, and Biggs et al.[30] measured left lane drift, which was defined as the percentage of the vehicle range that exited the road, to measure performance. These previous studies showed different results.

As mentioned above, one similarity with the preceding study is that the study outcome of lane maintenance was used to measure driving ability. However, the test time of driving was longer than that used in our study. In the present study, the examination time of driving tests was 5 min, and the total time was 15 minutes. However, other studies have measured driving performance during long driving time ranging from one hour to four hours. Thus, we speculated that the differences in SDLP between our study and previous studies were also due to the test time.

The limitations of the study were as follows: 1) we did not evaluate the participants’ habits of daily caffeine intake, 2) the washout time was not adequate, 3) we did not evaluate the influence of caffeine withdrawal, and 4) we did not evaluate the another cognitive function, also strictly linked to driving performance such as visuospatial ability[28] and semantic fluency[36]. A further study that considers these points is necessary to confirm our preliminary results. In conclusion, a single instance of caffeine intake changed some neurocognitive functions and driving performance in healthy volunteers.

## Supporting information

S1 File Consort checklist. (PDF)

S2 File Clinical trial protocol. (PDF)
