# Supplementary material for: Effect of single caffeine intake on neuropsychological functions in healthy volunteers: A double-blind placebo-controlled study
Source: PLoS One. 2018 Oct 31;13(10):e0202247. doi: 10.1371/journal.pone.0202247 (PMC6209127; doi:10.1371/journal.pone.0202247)
Supplement: S2 File — (PDF) [file pone.0202247.s002.pdf]

（様式第２号）

審　査　結　果　通　知　書

平成 27 年 12 月 9 日

申請者
精神医学　阿竹　聖和　殿

産 業 医 科 大 学
学 長　東　　敏　昭　印

受付番号　第 H27-184 号

課 題 名：カフェインが運転に及ぼす影響とその機序

研究者名：阿竹　聖和　吉村　玲児　香月　あすか　小西　勇輝　井形　亮平

上記課題の実施の可否については、平成２７年６月３日の産業医科大学倫理委員会の審査結果を踏まえ、下記のとおり決定したので通知します。

記

決定内容	（承認）　条件付承認　変更の勧告　不承認　非該当　その他
承認附帯条件	1　実施事項等の対象となる者の同意書の写しは、研究実施責任者が責任をもって保管する。<br>2　実施結果等は、速やかに報告すること。<br>なお、一定期間経過観察を必要とするものについては、適宜経過報告を行うこと。
条件・変更・不承認の理由	

Notification of examination outcome

December. 9, 2015

Applicant
Department of Psychiatry
Kiyokazu Atake

University of Occupational and Environmental Health
President Toshiaki Higashi

Receipt number: H27-184

Study title: Effect of single caffeine intake on neuropsychological functions.

Researcher: Kiyokazu Atake, Reiji Yoshimura, Asuka Katsuki, Yuki Konishi, Ryohei Igata

Decision based on the examination outcome of the University of Occupational and Environmental Health ethics panel in Jun. 3, 2015 was given below.

Decision	Accept
Subject to approval	1. Responsible person of study implementation will maintain records of copy of consent document.<br>2. You must report an implementation result promptly.
Reason of unapproving	

I certify that the copy foregoing is a correct translation.

Hikaru Hori (Medical doctor)
3rd. July 2017

（様式第１号）

倫理審査申請書

H27 年　4 月　30 日

産業医科大学学長　殿

所　　属　　精神医学教室
職　　名　　助教
申請者名　　阿竹　聖和　　　印
所属長名　　吉村　玲児　　　印

※受付番号　第　　　　号

１　課題名
カフェインが運転に及ぼす影響とその機序

２　実施責任者

所属	職名	氏名
医学部精神医学	助教	阿竹　聖和

３　実施分担者

所属	職名	氏名
医学部精神医学	教授	吉村　玲児
医学部精神医学	助教	香月　あすか
医学部精神医学	産業医学修練医	小西　勇輝
医学部精神医学	産業医学修練医	井形　亮平

４　実施事項等の概要

カフェインは茶やコーヒー等の様々な食品や飲料水に含まれており、車の運転や業務、学習などの効率を上げる目的で、コーヒーを飲用する一般的慣習がある。カフェインを摂取することによって記憶の定着率の上昇や（Nature Neuroscience 2014）、意思決定（Psychopharmacology 2014）といった認知機能を強化するという報告されている。

2013 年の交通事故発生状況は 629,021 件であり（2013 年警察庁交通事故統計）、交通事故の要因は、ドライバーの「認知・判断ミス」が 93％を占め、様々な認知領域が大きく関与していることが示唆される。商業用自動車の長距離ドライバーにとって、カフェイン添加物を摂取している人は、事故のリスクを 63％減少するという報告もある。（British Medical Journal 2013）。カフェイン添加物を摂取することが運転の敏捷性維持の補助戦略として有用である可能性があるが、カフェインの運転遂行能力や認知機能に及ぼす影響や、適切な摂取量・摂取時間に対しては明らかとなっていない。

今回の研究では、これまでに研究されたことのないカフェインと認知機能、運転遂行能力の関係を無作為割り付け試験（RCT）で検証する。患者背景、睡眠効率、活動量、各種バイオロジカルマーカーを解析・同定することで、カフェインの運転遂行能力や認知機能に対する影響を多角的に検証し、労働効率の上昇や、事故の減少など、社会経済や労働衛生に貢献することを目標とする。

５　実施計画（対象、期間、場所）

対象：健常人を対象とし、以下の基準を満たしたものとする。

① 20 歳以上 60 歳未満、男女を問わない
② 「精神障害の診断と統計の手引き」の研究における健常者用の構造化面接法（SCID-I-RV/NP : The Structured Clinical Interview for DSM-IV Axis I Disorders, Research Version、Non-patient Edition）を用いて健常者と確認された者

③　第一種運転免許を有する者
④　文書にて自由意思による同意が得られた者

予定症例数：　100 症例
期 間：平成 27 年 7 月～平成 30 年 6 月
場 所：産業医科大学　精神医学教室
募集方法：学内掲示

方法：

上記を対象に、睡眠覚醒リズム表を用いて 7 日間の睡眠の自己評価を行う。同時に、アクチグラフ（腕時計タイプ）を用いて活動性、睡眠効率、生活リズムの他覚的評価も行う。3 日間のカフェインを含有する飲料水や食品を摂取せず、飲料水は水飲みとする期間（ノンカフェイン期間）を設ける。患者背景を聴取した上で、封筒法により、カフェイン 200mg の錠剤を摂取する群と、プラセボ群のいずれかの群に無作為に割り付ける。試験開始前とカフェイン摂取した約 2 時間後に採血 20ml を行い､血漿 HVA(homovanillic acid)濃度や血漿 MHPG(3-methoxy-4-hydozyphenylglycol)濃度、血清 BDNF（Brain-derived neurotrophic factor）濃度、血清カフェイン濃度を検討する。

試験開始 2 か月前（測定前には 3 日間のノンカフェインの期間を設ける）と試験時にドライビングシミュレータを用いての運転遂行能力を測定し、認知機能検査として Trail maing test(TMT)、レイ複雑図形検査、かなひろいテスト、Tower of London test、n-back test、Continuous Performance Test(CPT)をカフェイン摂取後に行う。検査に要する時間は計 1 時間程度である。

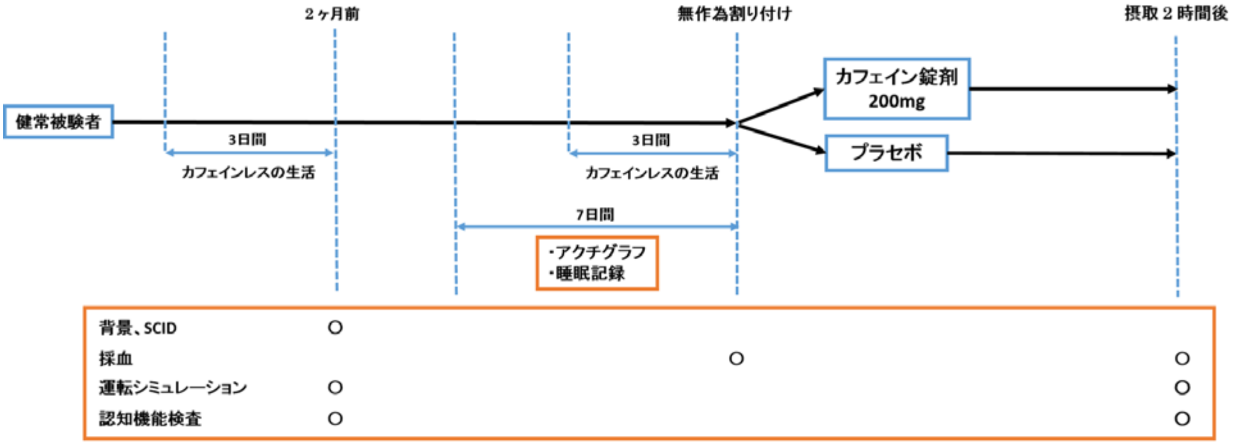

6　実施事項等における倫理的配慮について

（1）被験者の自由な選択の保障

被験者は、本研究に参加することの利益と不利益とを説明された上で、参加または不参加を自由に選択できる。また、参加の意思決定がなされた後においても、いつでもその同意を撤回することが出来る。さらに、不参加又は途中で参加を中止した場合でも不利益な扱いを受けないことが保障されること等が、担当者より被験者に十分に説明される。

（2）被験者のプライバシー確保に関する対策

研究データは、被験者が特定できないように、被験者番号を付して、連結可能匿名化し、その番号と実名の対応表とともに本学精神医学教室の鍵のかかる保管庫に保管する。この研究によって得られた成果を学会や論文などに発表する場合には、個人を特定できる氏名、住所などの個人情報は一切使用しない。廃棄は研究終了後、匿名化を確認した後、直ちに行う。研究参加の同意を撤回した場合もその時点までに得られた個人情報の匿名化を確認のうえ、直ちに廃棄する。

（3）対象者に理解を求め、同意を得る方法

本研究の意義、目的、方法、被験者が被りうる不利益と危険性に関する説明文書を作成し、文書及び口頭にて十分な説明を行い、同意書への署名を依頼する。

（4）研究結果の被験者への開示について<br>研究結果は原則非開示とするが、被験者が希望すれば、本人の結果を開示する。また、全体のデータは学会などで公表後、請求に応じて開示する。尚、データを学会等で公表する場合、個人を特定できる氏名、住所などの個人情報は一切使用しない。<br>（5）被験者から採取した生体試料の取り扱いについて（保管・廃棄方法など）<br>採取した生体試料は、本研究に使用するまで、本学精神医学教室研究室の冷蔵庫に保存する。研究終了後、生体試料は研究責任者の管理のもと、匿名化を確認し直ちに廃棄する。<br>また同意を撤回した場合にも、その時点までに得られた生体試料、個人情報及び研究結果を、同様の措置にて廃棄する。<br>（6）被験者から採取した生体試料の目的外利用について<br>採取した生体試料は本研究の目的以外には使用しない。
7　考えられる被験者への利益、危険性及び不利益並びにそれらが生じた場合の措置方法<br>今回の研究で用いた検体の実験結果から提供者に直接の利益は生じないが、研究で行った実験の結果として、カフェインの適切な摂取方法や、運転遂行能力の新たな評価方法が確立されれば、将来においてその新規技術により、医療現場だけでなく産業現場にも応用出来る可能性はある。本研究では、カフェイン摂取量は 200mg と一般的な缶コーヒー1～2 杯分であり、被験者への危険性はほとんどないものと考えられる。尚、もし気分不良などの訴えなどの不測の事態が生じた場合には、直ちに中止して適切な処置をとる。また、個人情報が漏洩した場合、この研究結果の情報により社会的に不当な扱いを受ける可能性があるので、研究実施責任者によって被験者番号を付して、連結可能匿名化し、情報の徹底管理によって漏洩を防止する。また研究成果の公表の際には個人が特定できないように措置をする。
8　医学からみた客観的意義<br>カフェインを摂取することによって、認知機能・運転遂行能力が高まることが予想される。コーヒーの飲用などによるカフェイン摂取という簡易な方法で、労働者における労働効率の向上や死亡事故・交通事故の減少への寄与が期待される。
9　研究の費用<br>本研究の実施に関わる費用は、精神医学教室の研究費から充当され、被験者への費用負担はない。また研究参加に対する謝礼はない。
１０　緊急時の連絡先<br>研究実施責任者：産業医科大学精神医学教室 助教 阿竹 聖和 093-603-1611(PHS 7369)<br>研究実施担当者：産業医科大学精神医学教室 産業医学修練医 小西 勇輝 093-603-1611(PHS 7294)<br>休日や夜間で研究班員が不在の場合には神経・精神科の当直医が班員に連絡する。
１１　その他<br>本研究の成果に基づいて、特許権などの知的財産権が生じる可能性もあるが、生体データ提供者である被験者には知的財産権は発生しない。

注：※印は記入しないこと

Clinical Trial Protocol

Objective:

This study is conducted in a double-blind, randomized, placebo-controlled manner from January 2016 to December 2016 (Fig 1). The participants are randomly assigned to either the caffeine group (N = 50) or the placebo group (N = 50) via the envelope method. The protocol (S1 file) is conducted as follows. First, individuals don't consume caffeine for 3 days prior to the study. Participants are randomly assigned to receive 200-mg doses of caffeine or a placebo. Blood samples are obtained before and 120 minutes after the administration of caffeine or a placebo. The plasma caffeine concentration is measured via high-performance liquid chromatography (HPLC). Thirty minutes after administration, the cognitive functions of participants are evaluated with the Symbol Digit Coding Test (SDC), the Stroop Test (ST), the Shifting Attention Test (SAT) and the Four Part Continuous Performance Test (FPCPT) using Cognitrax, a software program used to test neurocognitive functions. After the cognitive function tests are conducted, the driving performance of participants is evaluated using a driving simulator. Main outcome measure is driving performance before and after the administrations of caffeine or a placebo. Secondary outcome measures are caffeine concentrations and cognitive functions.

Subjects

Included criteria

1) Physically healthy, 2) without any psychiatric diseases via the Structured Clinical Interview for DSM-IV (SCID), 3) have a Japanese driver's license.

Excluded criteria

1) Heavy caffeine consumers, 2) smokers, 3) taking any medications.

Setting a sample number

One hundred participants are setting according to the power analysis. Duration of the present study is from July 2015 to June 2018.

Method

Plasma preparations

Whole blood samples are collected in heparinized tubes and centrifuged, and plasma are stored under -20 C.

Analysis of plasma caffeine levels

Plasma caffeine levels are analyzed using HPLC (Agilent 1220 Infinity II LC, Agilent Technologies, Inc. United States).

Evaluation of driving ability

Harsh-braking test and Road-tracking test are performed using a driving simulator (UC Win/Road Driving Simulator, FORUM8 Co., Ltd. Japan).

Evaluation of cognition

Cognitive function is assessed with the CNS Vital Signs (CNSVS) (CNS Vital Signs LLC, Morrisville, USA) computerized battery of tests.

Statistical analysis

Statistical analyses are performed using STATA software. Differences between the caffeine group and placebo group regarding demographics and baseline characteristics, driving ability and neurocognitive functions are assessed via non-paired t-tests and chi square tests. Repeated measures analysis of covariance is performed for blood caffeine concentration levels with the baseline data serving as the covariate. All statistical tests are two-tailed, and a P-value below 0.05 is considered significant.
